# Spontaneous hepatic hemorrhage as presentation of metastasized papillary thyroid carcinoma: a case report

**DOI:** 10.1186/s13256-024-04797-5

**Published:** 2024-09-23

**Authors:** Jacob Thomasson, Bodil Andersson, Lo Hallin Thompson, Caroline Williamsson

**Affiliations:** Department of Clinical Sciences Lund, Surgery, Lund University and Skåne University Hospital, 221 85 Lund, Sweden

**Keywords:** Spontaneous hepatic hemorrhage, Papillary thyroid carcinoma, Hepatic metastasis, Case report

## Abstract

**Background:**

Spontaneous hepatic hemorrhage is a rare condition, most commonly diagnosed in patients with hepatocellular carcinoma or hepatic adenomas, and is seldom caused by metastatic disease. In this case report, we present a patient with spontaneous hepatic hemorrhage due to hepatic metastasis of papillary thyroid carcinoma, an exceptionally rare occurrence.

**Case presentation:**

The patient was a 77-year-old white male with a history of atrial fibrillation treated with apixaban. He presented at a local hospital with abdominal pain and nausea. A CT scan revealed a hepatic lesion in segment 3 with an adjacent hematoma. He was referred to our tertiary center and treated conservatively. Further evaluation revealed an intrathoracic goiter containing a tumorous process diagnosed as a papillary thyroid carcinoma (PTC), and the patient subsequently underwent thyroidectomy. A biopsy of the hepatic lesion confirmed it as a PTC metastasis. Due to worsening abdominal pain and anorexia, the patient underwent subacute hepatic segmental resection. Postoperatively, he developed iodine-refractory disease with disseminated metastasis and passed away 22 months after the initial admission.

**Conclusions:**

To our knowledge, this is the first recorded case of metastasized papillary thyroid carcinoma presenting with spontaneous hepatic hemorrhage—adding to the list of rare causes for this condition.

## Background

Spontaneous hepatic hemorrhage (SHH) is a rare condition with multiple etiologies where hepatic lesions, either malignant or benign, are the most common underlying pathology [1]. In regions with high incidences of hepatocellular carcinoma (HCC), such as East and Southeast Asia and Northern Africa [2], HCC is the predominant cause [3]. Less common causes include hepatic metastases, other benign tumors, and connective tissue diseases [3, 4].

The underlying pathophysiological mechanisms are poorly understood but are most likely multifactorial, related to processes of the underlying condition. In tumors, these mechanisms include neoangiogenesis, which is susceptible to minor trauma or physiological changes in blood pressure, central tumor necrosis with associated hematoma, and rapidly growing lesions exerting pressure on the surrounding tissue, possibly enfeebled by cirrhosis, leading to either rupture or thrombotic congestion that increases pressure until structural failure occurs [3, 5].

The condition presents a multidisciplinary challenge for general surgeons, hepatobiliary surgeons, and interventional radiologists, requiring prompt and decisive measures tailored to the underlying disease and comorbidities. For hemodynamically stable patients without active bleeding, conservative management with adequate hospital surveillance is preferred. In patients with radiological signs of active bleeding, transarterial embolization should be considered as a first-line alternative to surgery [4, 6], with surgery being reserved for hemodynamically unstable patients or when endovascular therapy is unsuitable or has failed. Once hemostasis is achieved, further workup is recommended, and definitive treatment depends on the underlying pathology [4].

Papillary thyroid carcinoma (PTC) is the most common type of well-differentiated thyroid cancer (WDTC), accounting for approximately 85% of cases [7]. Although typical WDTCs are generally indolent tumors confined to the thyroid gland, 4–15% of patients develop distant metastases, most commonly in the lungs, followed by bone. Liver metastasis is rare, with a reported frequency of 0.5% in metastasized WDTC, and is usually synchronous with other distant metastases [8, 9]. In cases of metastatic disease, 10-year survival drops significantly from around 85–95% to 50% [9]. Treatment for metastatic disease is multifaceted and individualized, including radioactive iodine therapy, hormone suppression therapy, surgery, external radiation, and tyrosine kinase inhibitors [10].

In this article, we present a case of metastasized PTC that manifested as spontaneous hepatic hemorrhage—a combination of two rare conditions that, to our knowledge, has not been previously reported in the literature.

## Case presentation

**Fig. 1:**
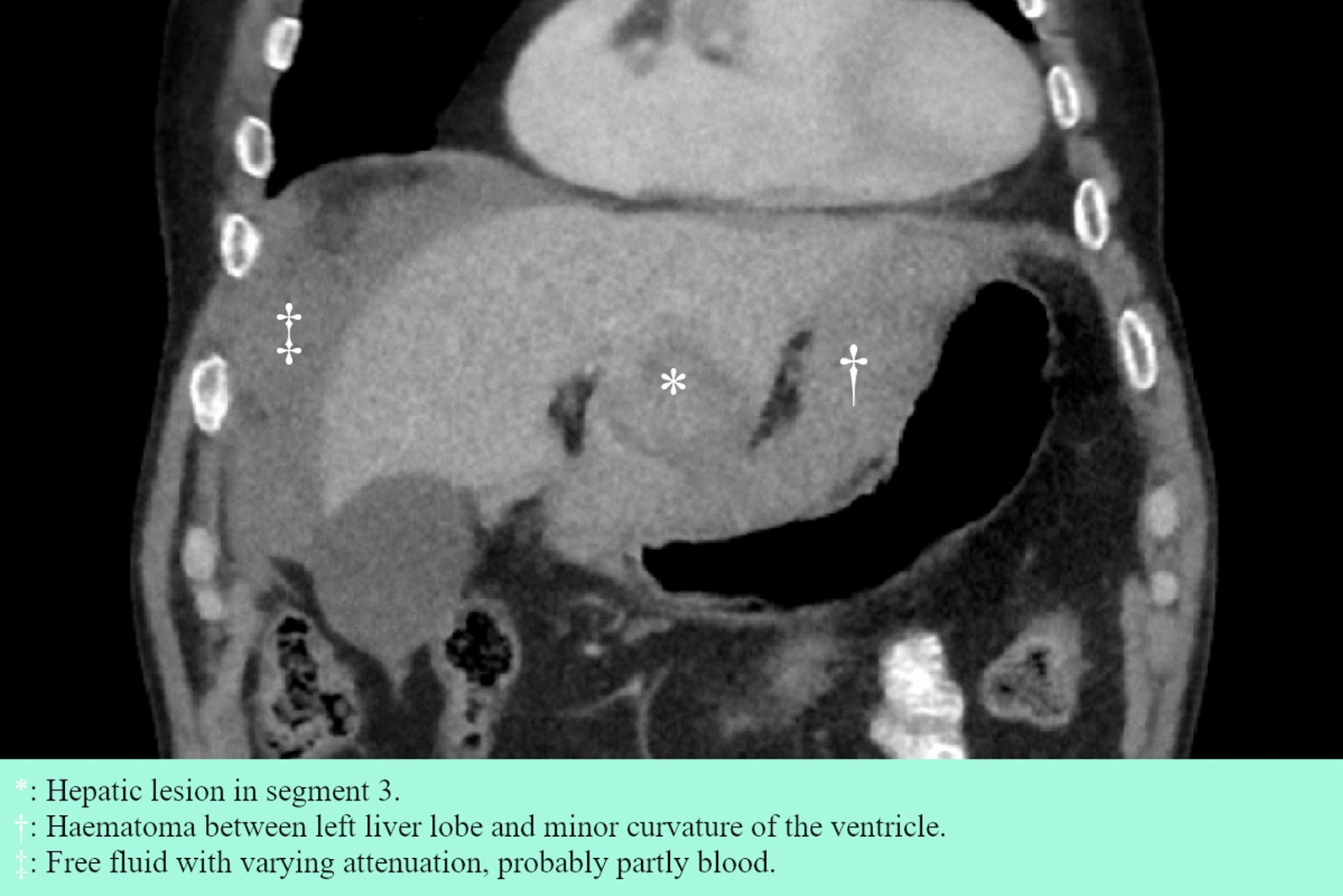
Coronal section of abdominal CT scan on the day of admission

**Fig. 2:**
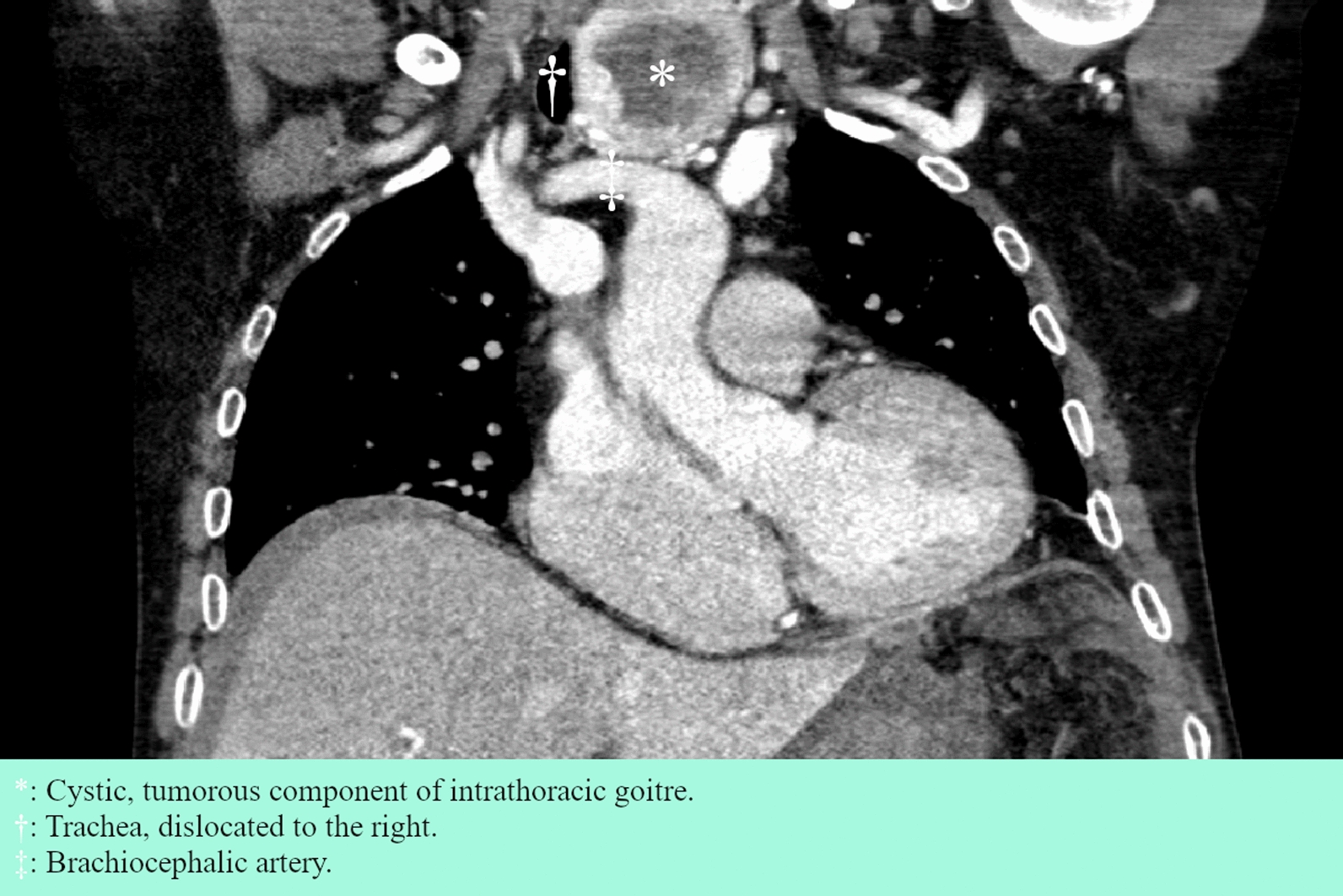
Coronal section of thoracoabdominal CT scan on the fourth day after admission

The patient was a 77-year-old white male with a history of atrial fibrillation treated with apixaban. He had a family history of a sister with an undefined central nervous system tumor diagnosed in her 20s. He presented to the emergency department of a local hospital with nausea and slow-onset abdominal pain that began approximately 10 hours before admission. Clinical examination revealed tenderness in the right and upper central abdomen. His vitals showed a blood oxygen saturation of 95% with 5 L/minute O_2_, irregular tachycardia at 120–140 beats per minute (bpm), stable blood pressure at 140/85 mmHg, and a temperature of 37.5 °C. Blood tests showed elevated leukocytes at 20.2 × 10^9^/L, C-reactive protein (CRP) at 43 mg/L, and hemoglobin concentration of 129 g/L. Suspecting cholecystitis or appendicitis, an abdominal computed tomography (CT) scan was performed, revealing a 5.5 cm rounded and slightly irregular mass in the third liver segment with a medial perihepatic hematoma and laterally free fluid with varying attenuation (Fig. 1). The condition was interpreted as a spontaneous rupture and hemorrhage of a hepatic lesion of unknown origin, possibly HCC. Assessment showed no obvious radiological signs of cirrhosis, bilirubin 13 µmol/L, albumin 26 g/L, and prothrombin time international normalized ratio (PT–INR) of 1.1, corresponding to Child–Pugh B in the presence of cirrhosis. The patient was referred and transported to the surgical ward at our tertiary center for observation, given the availability of endovascular therapy in case of deterioration. He received prothrombin complex concentrate and tranexamic acid while apixaban was discontinued. During observation, the patient remained hemodynamically stable, although hemoglobin levels dropped to a minimum of 100 g/L on the third day of admission before spontaneously rising without the need for transfusion. A follow-up abdominal and thoracic CT scan on the fifth day showed a slight expansion of the hematoma from 5 cm × 15 cm × 8 cm to 6.5 cm × 16 cm × 11 cm, as well as an intrathoracic goiter with a 5.5 cm cystic and irregular tumorous component (Fig. 2). An ultrasound of the neck revealed a 9 cm inhomogeneous, well-defined tumor with cystic elements corresponding to the European thyroid imaging reporting and data system (EU-TIRADS) 3, classifying it as a low-risk lesion [11]. Ultrasound-guided fine needle aspiration (FNA) was performed due to its size. Cervical lymph nodes were described as normal. A week after admission, the patient developed a fever and elevated CRP, for which he was treated empirically with piperacillin–tazobactam. He was discharged on the 12th day after admission, after cultures returned negative.

**Fig. 3:**
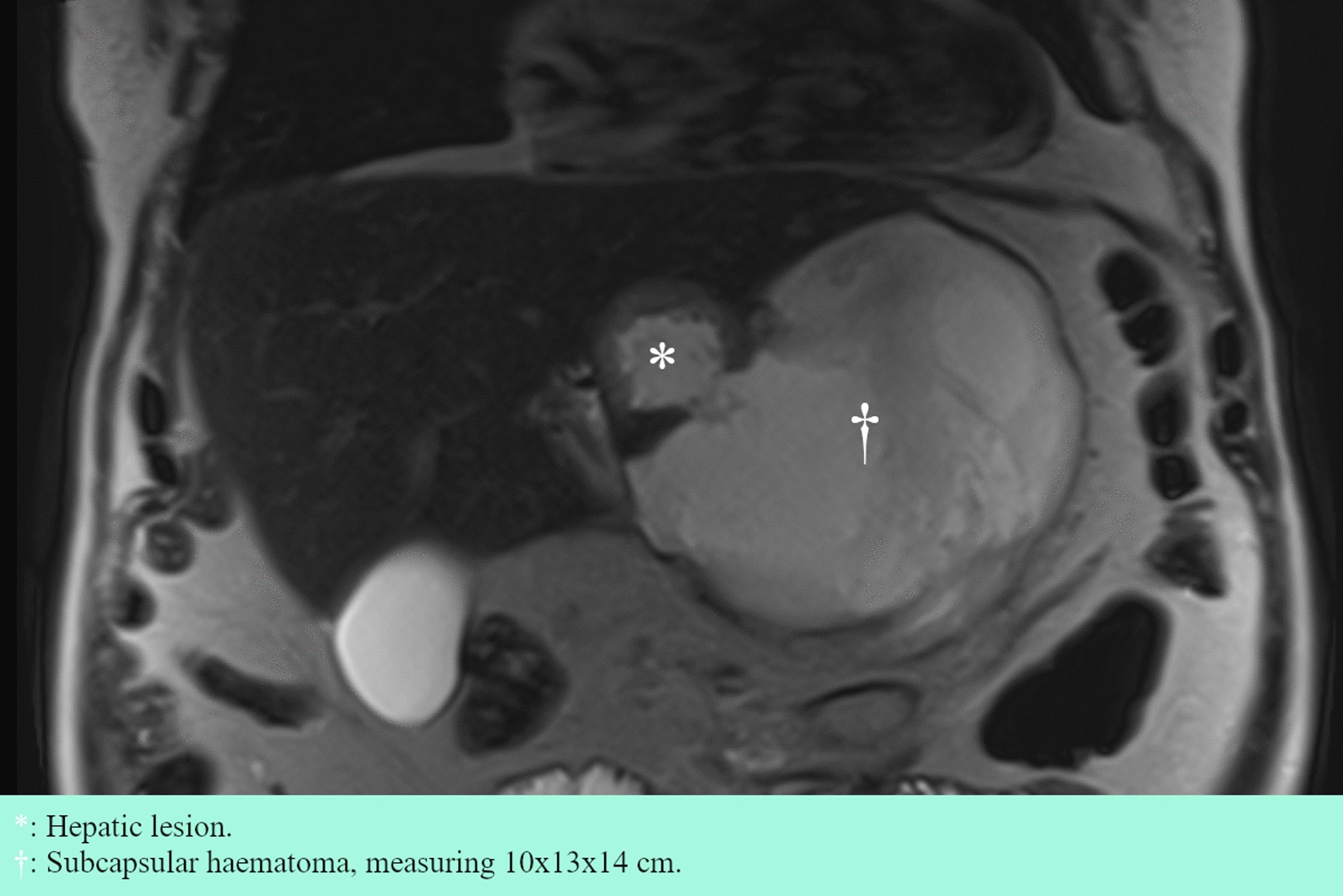
Coronal section of abdominal MRI, T2 weighted, 21 days after initial admission

Diagnostic workup continued on an outpatient basis with a magnetic resonance imaging (MRI) of the liver, which showed a cystic, thick-walled tumor of 4.5 cm in size in segment 3 with a surrounding 10 cm × 13 cm × 14 cm subcapsular hematoma (Fig. 3). The tumor was described as atypical for HCC and was suspected to be a metastasis. A core needle biopsy of the tumor was recommended.

In the meantime, FNA biopsies of the thyroidal mass suggested PTC, with Bethesda 5 [12]. Endocrine surgeons recommended total thyroidectomy, which the patient underwent uneventfully, with no intraoperative signs of local node metastases. He was discharged on postoperative day (POD) 1. Results from the core needle biopsy of the hepatic lesion were not yet available at the time of surgery.

**Fig. 4:**
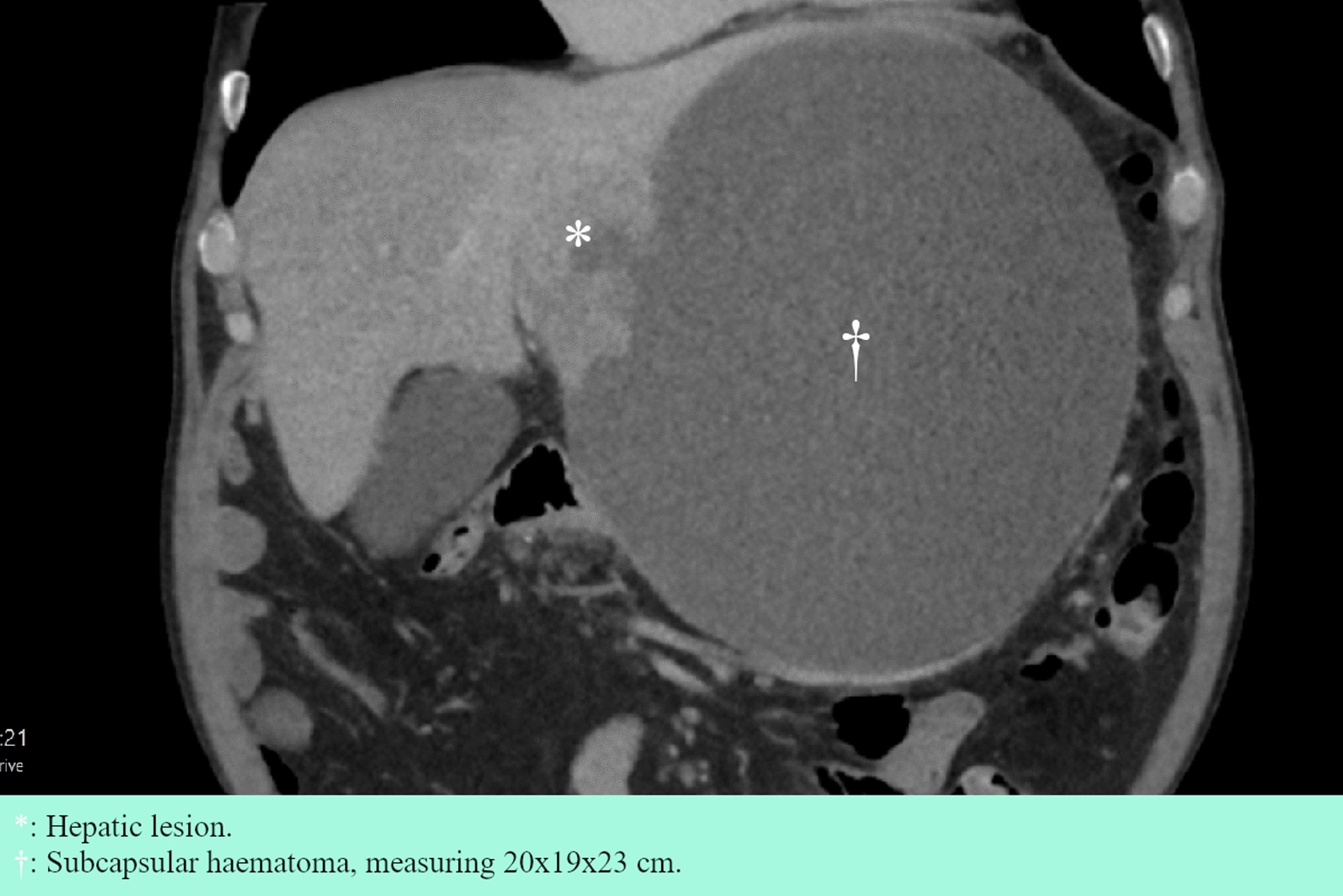
Coronal section of abdominal CT scan, 3 months after initial admission

Approximately 2 weeks post-thyroidectomy, results from the core needle biopsy arrived, indicating PTC metastasis. The endocrine surgeons were contacted again, and radioactive iodine therapy (RAI) was considered. However, the patient reported abdominal pain, abdominal swelling, anorexia and general fatigue, which had exacerbated over the past month. An urgent abdominal CT scan showed progression of the perihepatic hematoma to 20 cm × 19 cm × 23 cm (Fig. 4). The patient was admitted and underwent subacute resection of the left lateral segment, 16 days post-thyroidectomy. Intraoperatively, biopsies were taken from three suspected nodules on segment 4B, the omentum, and the diaphragmatic peritoneum. The patient experienced postoperative delayed gastric emptying (DGE) and a suspected infection of unknown origin, treated briefly with a nasogastric tube and piperacillin–tazobactam, respectively. He was discharged on POD 7 and resumed apixaban treatment.

**Fig. 5:**
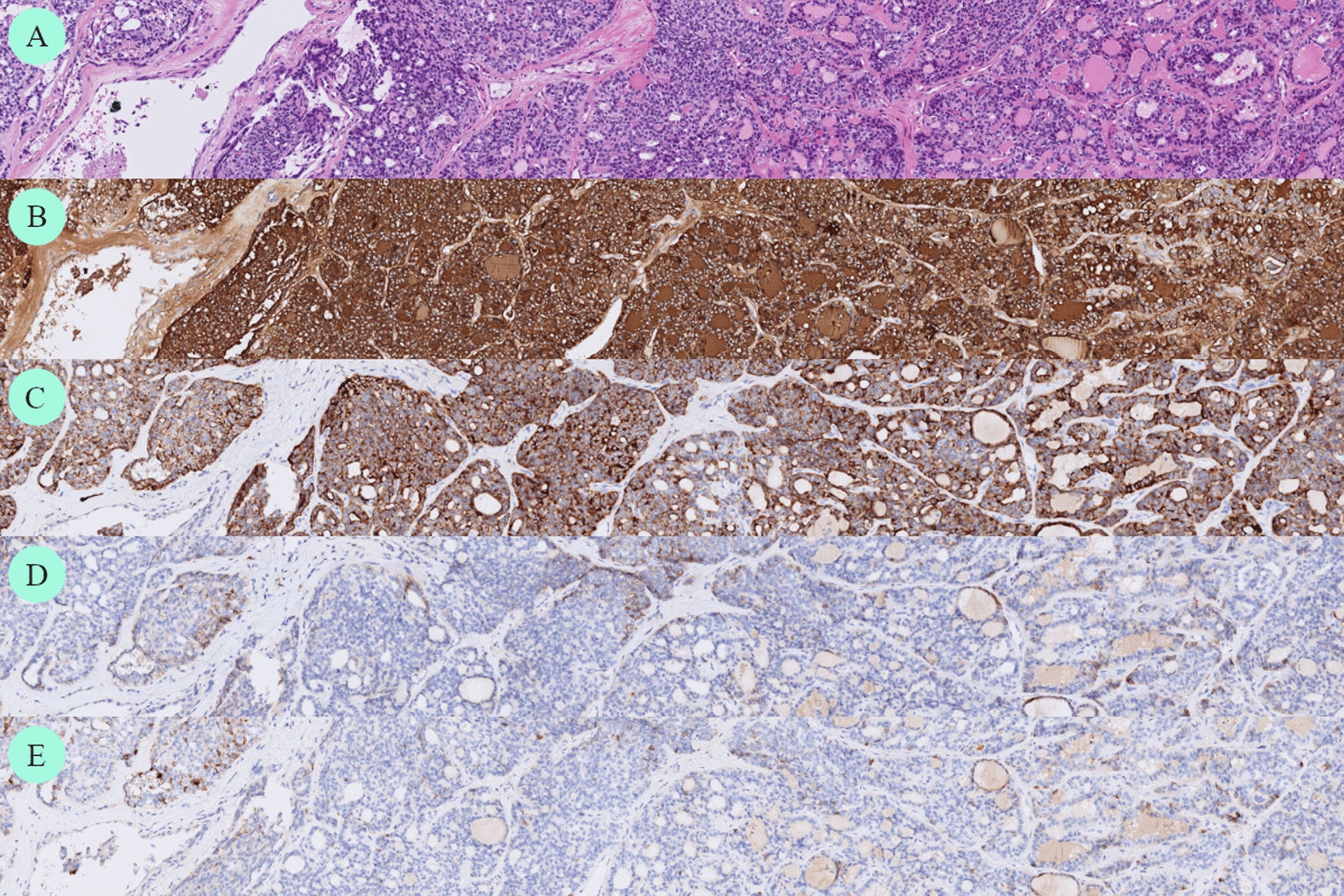
Histopathology of thyroid lesion. Original magnification × 40. Hematoxylin–eosin (H&E) routine stain (**A**). Positive immunohistochemical stain for thyroglobulin (**B**) and CK19 (**C**). Negative immunohistochemical stain for CD56 (**D**) and TPO (**E**), typical for PTC

**Fig. 6:**
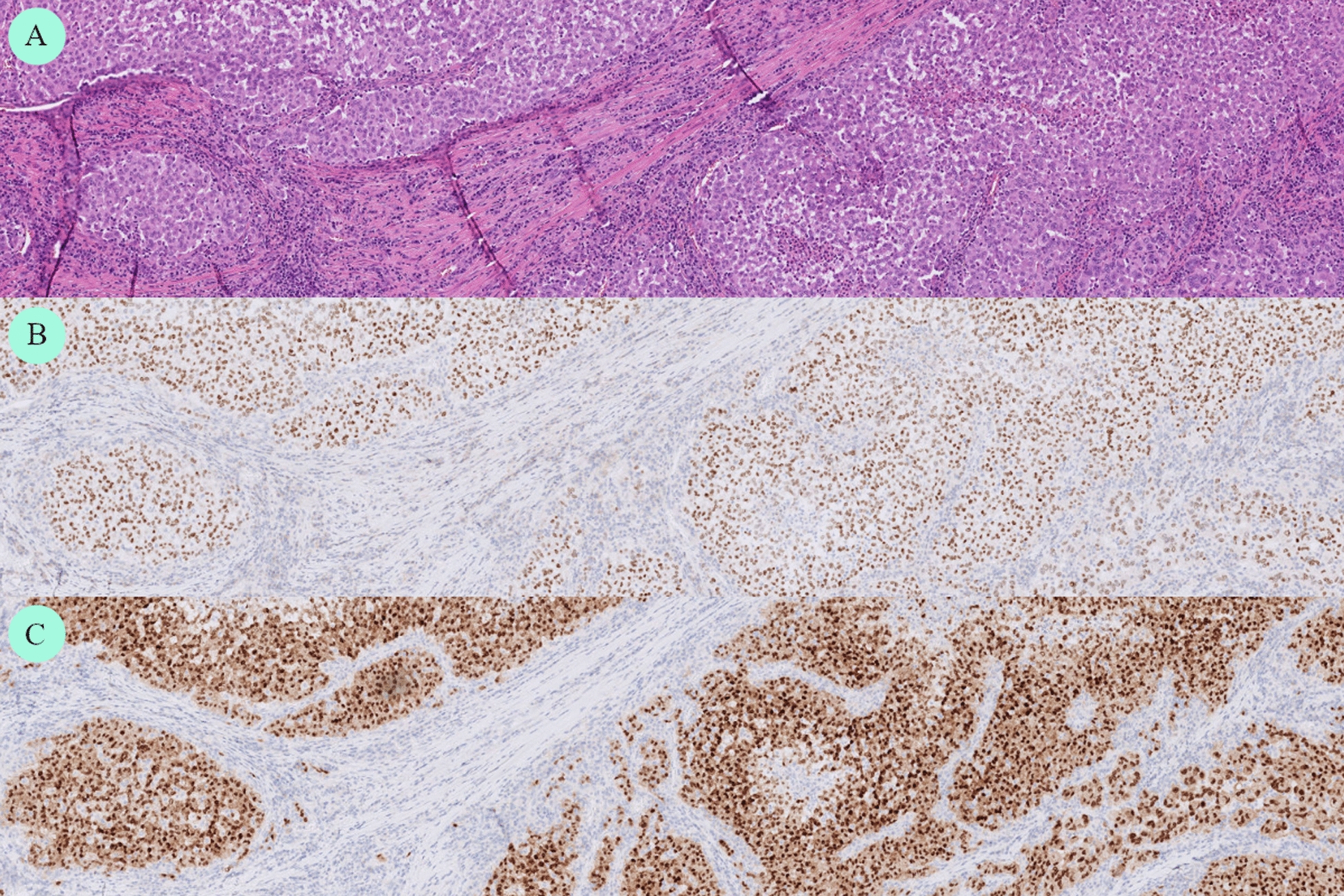
Histopathology of hepatic lesion. Original magnification × 40. H&E routine stain (**A**). Positive immunohistochemical stain for TTF1 (**B**) and PAX8 (**C**), indicating thyroid follicular differentiation and thus metastasis

Pathological examination (Fig. 5) of the thyroid showed a 9 cm classic variant PTC with a follicular growth pattern, minimal extrathyroidal invasion into fibrous tissue, and intravascular invasion. A lymph node located above the isthmus contained a 2.5 mm metastasis, and the tumor was staged as pT3N1aM1. Pathological examination following the liver resection (Fig. 6) revealed two PTC metastases, 6 and 4 cm in size, and a PTC metastasis in the diaphragmatic lesion. Adjuvant RAI totallng 3.7 Gbq, was recommended.

The patient was referred to the oncology department and underwent RAI. Follow-up scintigraphy showed remaining uptake in the cervical area, and elevated thyroglobulin levels were detected, indicating residual disease. A 6-month follow-up with ultrasound of the neck and liver was planned.

Approximately a month before the planned follow-up, the patient developed macroscopic hematuria and experienced abdominal and back pain. Apixaban was again discontinued. Ultrasound of the liver revealed two suspected liver metastases, 2 and 2.5 cm, respectively. A fluorodeoxyglucose positron emission tomography (FDG–PET) was subsequently ordered, which showed focal hypermetabolism in the right lobe, approximately 2.5 cm in size, as well as in the vertebrae T11, L2, and L5. A soft-tissue component was noted infiltrating the spinal canal at the T11 level. MRI confirmed intraspinal growth at the T11 level, although no neurological deficits were observed. The patient was referred for radiation treatment, receiving 16 Gy in two fractions.

Cystoscopy confirmed two tumorous processes in the bladder, and the patient underwent a transurethral resection of bladder tumor (TURBT). Microscopic analysis identified PTC metastasis and a papillary urothelial carcinoma graded G1pTa.

The patient was diagnosed with iodine-refractory disease and was scheduled to begin treatment with lenvatinib, a tyrosine kinase inhibitor. However, this treatment was postponed due to the TURBT procedure. A CT scan performed 1 week into lenvatinib treatment showed progression of the liver metastases, along with new suspected peritoneal and splenic metastases, and enlargement of cardial lymph nodes. The patient experienced debilitating urge incontinence and back pain. Due to the presence of postoperative lesions in the bladder, lenvatinib was discontinued for a month, and the patient received an additional 8 Gy of radiation therapy for the vertebral metastases.

At the 3-month follow-up, there was slight disease progression, leading to the initiation of zoledronate and the patient was reportedly feeling better. At a subsequent follow-up 3 months later, a CT scan showed no progression, and thyroglobulin levels had slightly decreased. However, the patient was diagnosed with a pulmonary embolism and resumed apixaban. Approximately 3 months later, the patient’s condition deteriorated, with increasing general fatigue and back pain. A CT scan revealed extensive tumor progression, including lung metastases. Lenvatinib was discontinued and palliative care was initiated. The patient passed away approximately a month later, 22 months after the initial admission.

## Discussion

To our knowledge, a case of SHH due to PTC metastasis has not been previously reported in the literature. While SHH cases resulting from liver metastases have been documented for a variety of primary tumors [3], it remains highly unusual compared with bleeding associated with HCC or hepatic adenoma. Distant metastases in WDTC are rare, especially in PTC, where the prevalence is reported to be around 5%, in contrast to follicular and Hurthle cell carcinomas, where it is more common [13]. Furthermore, isolated hepatic metastasis in WDTC is exceedingly rare, as hepatic metastases typically present synchronously with disseminated disease [8, 9]. This combination led us to initially believe that there was no correlation between the hepatic and thyroid lesions, resulting in separate investigations and treatment until pathological analysis was complete. This highlights both the importance and the challenges of a structured, multidisciplinary approach to the diagnosis and treatment of rare conditions.

From a surgical perspective, the treatment of the patient’s SHH was consistent with current recommendations [4]. The patient was promptly transferred to our tertiary center to ensure availability of endovascular therapy and hepatic surgeons. Initial hemostasis was achieved conservatively, followed by diagnostics to identify the underlying lesion. Although SHH is rare, it is associated with high mortality in patients with HCC [4] and has been reported to account for approximately 1% of admission to hepatobiliary units [3], underscoring the importance of understanding its etiology and treatment. While transarterial embolization is an important and less invasive first-hand alternative to surgery in the treatment of SHH [4], it was not implemented in this case. It could be speculated that transarterial embolization might have played a role in reducing the expansion of the hematoma and the related pressure symptoms that ultimately prompted surgery. However, due to the hemodynamically stable nature of the hemorrhage and the absence of visible extravasation on imaging studies, this procedure was not performed during the initial admission. When the patient was readmitted, endovascular treatment would not have alleviated the pressure symptoms, which were his primary concern, and thus surgery was performed. Additionally, while surgical treatment is recommended for recurrent or iodine-refractory oligometastatic WTDC [10], this case exemplifies a situation where surgery was performed subacutely, before RAI therapy, due to severe symptoms.

Regarding the patient’s anticoagulant therapy with apixaban, it is reasonable to suggest that direct-acting oral anticoagulants (DOACs) can sustain and complicate the cessation of hemorrhage in SHH. However, there is no evidence indicating a higher risk or worse prognosis for SHH in patients receiving anticoagulant therapy with low-molecular weight heparin or vitamin K antagonists [1]. As the use of DOACs becomes more widespread, future studies will need to determine if this holds true, especially considering that reversal therapy is currently expensive and not available for all substances.

The patient ultimately developed iodine-refractory disease with metastases spreading to multiple organs, indicating aggressive tumor biology, which in contrasts with the typical indolent nature of PTC. Spontaneous rupture and subsequent hemorrhage of malignant hepatic tumors, the overwhelming majority being HCC, negatively impact prognosis. SHH poses a surgical emergency with a short-term mortality between 25% and 75% [4] and, in HCC cases, decreases both overall survival and disease-free survival in patients with resectable disease [14] . It is possible that the prognosis of our patient was negatively affected by the rupture of his hepatic metastasis due to tumor seeding, but given the aggressive and generalized metastatic pattern, it can also be argued that tumor biology played a more significant role.

While this case illustrates a combination of rare conditions that most clinicians will never encounter, it serves as a reminder of the importance of maintaining an open mind toward implausible explanations for poorly studied conditions due to their rarity. For the hepatobiliary surgeon, who may encounter several cases of SHH during their career, this case adds to the understanding that, although rare, hepatic metastases can present with spontaneous hemorrhage, necessitating thorough work-up before treatment for the underlying lesion is initiated.

## Conclusions

To our knowledge, this case report represents the first documented instance of spontaneous hepatic hemorrhage due to metastasized papillary thyroid carcinoma. It underscores the importance of comprehensive multidisciplinary diagnostics of the underlying hepatic lesion and contributes to the collection of rare causes for this condition.

## Data Availability

Not applicable.

## Abbreviations

SHH: Spontaneous hepatic hemorrhage
HCC: Hepatocellular carcinoma
PTC: Papillary thyroid carcinoma
WDTC: Well-differentiated thyroid cancer
CRP: C-reactive protein
PT–INR: Prothrombin time international normalized ratio
CT: Computed tomography
FNA: Fine needle aspiration
MRI: Magnetic resonance imaging
DGE: Delayed gastric emptying
POD: Postoperative day
RAI: Radioactive iodine therapy
FDG-PET: Fluorodeoxyglucose positron emission tomography
TURBT: Transurethral resection of bladder tumor
